# High-Pressure Design of Advanced BN-Based Materials

**DOI:** 10.3390/molecules21101399

**Published:** 2016-10-20

**Authors:** Oleksandr O. Kurakevych, Vladimir L. Solozhenko

**Affiliations:** 1 IMPMC, UPMC Sorbonne Universités, UMR CNRS 7590, MNHN, IRD UMR 206, Paris 75005, France; oleksandr.kurakevych@impmc.jussieu.fr; 2 LSPM–CNRS, Université Paris Nord, Villetaneuse 93430, France

**Keywords:** boron nitride, phase diagram, advanced materials, high pressure

## Abstract

The aim of the present review is to highlight the state of the art in high-pressure design of new advanced materials based on boron nitride. Recent experimental achievements on the governing phase transformation, nanostructuring and chemical synthesis in the systems containing boron nitride at high pressures and high temperatures are presented. All these developments allowed discovering new materials, e.g., ultrahard nanocrystalline cubic boron nitride (nano-cBN) with hardness comparable to diamond, and superhard boron subnitride B_13_N_2_. Thermodynamic and kinetic aspects of high-pressure synthesis are described based on the data obtained by in situ and ex situ methods. Mechanical and thermal properties (hardness, thermoelastic equations of state, etc.) are discussed. New synthetic perspectives, combining both soft chemistry and extreme pressure–temperature conditions are considered.

## 1. Introduction

**Figure 1:**
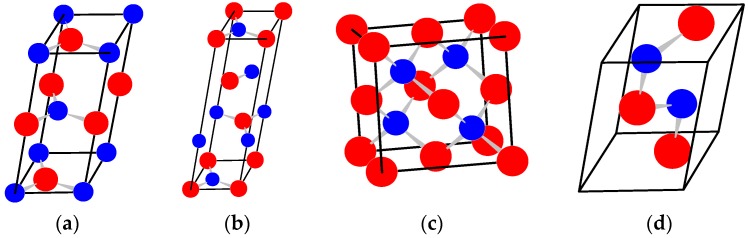
Crystal structures of BN polymorphs: (**a**,**b**) graphite-like hexagonal (hBN, 2H) and rhombohedral (rBN, 3R) low-density BN polytypes; and (**c**,**d**) cubic/sphalerite (cBN, 3C) and hexagonal/wurtzite (wBN, 2H) dense BN polytypes. The crystallographic axes are oriented in traditional way (**u**_x_ × **u**_y_ || **u**_z_), and the coordinate origin (0; 0; 0) is in the front-left-bottom corner.

Boron nitride (BN) was first synthesized in the mid-nineteenth century in hexagonal form (hBN, Figure 1a) [1], and a century later became a commercial product widely employed as powder (e.g., a lubricant or an additive to cosmetic products) and ceramic (e.g., pure pyrolytic BN produced by high-temperature CVD method is employed for furnace, electrical, microwave, and semiconductor components). Layered BN has several polytypes, the most famous, after hBN, is rhombohedral rBN (Figure 1b).

Cubic boron nitride with sphalerite structure (cBN, Figure 1c) was synthesized at high pressures and high temperatures in 1957, four years after the first synthesis of artificial diamond, and was considered as the second-to-diamond superhard phase [2]. Currently, cBN is produced on commercial scale. Another dense BN polytype, metastable boron nitride with wurzite structure (wBN) can be synthesized as a fine (~100 nm) powder by shock-wave compression of hBN. However, the use of wBN in cutting tools is limited by its low thermal stability [3]. All this became possible due to the development of new apparatuses and procedures, and was a real breakthrough in synthesis of artificial superhard materials, showed the possibility to use high-pressure technique in industry, and stimulated interest in this problem. Since that time a number of novel superhard high-pressure phases have been discovered, i.e., orthorhombic γ-B_28_ [4,5] and pseudo-cubic t’-B_52_ [6] boron allotropes, diamond-like BC_5_ [7] and cubic BC_2_N [8]. Thus, boron, carbon and nitrogen still remain the key elements for the hardness [9].

Design of new BN materials is aimed at: (i) superhard materials of high strength and wear resistance with thermal and chemical stability superior to diamond; (ii) high-performance composites with adjustable thermal and electrical conductivities [10]; (iii) new p- and n-type semiconductors [11]; and (iv) multiple challenging explorative applications as hydrogen-storage [12], 2D flexible nanoelectronics [13], optoelectronic and other materials [14,15,16]. The traditional solutions for design of advanced materials imply: (1) novel crystal structures and/or compositions; (2) low-dimensional materials; and (3) nanostructures. This approach was quite successful for boron (crystal structure [4,5] and composition [17,18]), silicon (crystal structure [19] and nanostructuring [20]), carbon (nanostructuring [21], composition [7]), etc. In the case of boron nitride, the major recent achievements are nanostructures created by both high-pressure technique [22] and soft chemistry [14].

In the present review, we highlight some recent achievements in high-pressure design of new advanced BN-based materials. The main routes to novel materials are nanostructuring by direct phase transformation and chemical interactions in the B–BN system under high pressures and high temperatures. Thermodynamic and kinetic aspects of such design is also discussed.

## 2. Advanced BN Precursors

The precursors for high-pressure synthesis are usually powders or pyrolytic bulks. In the case of (nano)powders, it may be quite difficult to assure the (surface) purity since, in air, hydrolysis occurs—although slowly—even at ambient conditions. Here, we will discuss only precursors important for creation of new materials.

### 2.1. Pyrolytic BN

**Figure 2:**
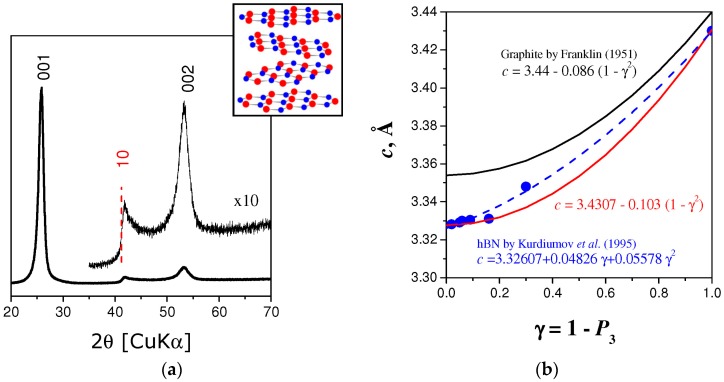
Crystal structure of turbostratic BN described in the framework of the random-layer lattice model: (**a**) Typical powder diffraction pattern with approximately symmetric 00*l* reflections and strongly asymmetric two-dimensional *hk* reflections (with displaced maximum), *insert*: randomly displaced and rotated BN layers forming turbostratic structure; (**b**) Interlayer distance of turbostratic BN samples with different degree of turbostratic disorder.

Pyrolytic BN (sometimes noticed as pBN) is characterized mainly by the method of synthesis (high-temperature CVD) rather than by a particular crystal structure [23]. Depending on the synthesis conditions (e.g., temperature) it can have turbostratic, hexagonal or rhombohedral structures (tBN, hBN or rBN, respectively). However, often thus obtained phases have partially ordered structures, also called mesographitic. The crystal structure is one of the crucial factors for the properties of resulting BN material. The principal element of pBN is a hexagonal BN borazon layer typical for all “graphitic” polymorphs (Figure 1a,b, Inset of Figure 2a). Geometrically, the crystallite can be imagined as a prism with layers parallel to the base stacked along the crystallographic *c*-axis (in general case, *c* corresponds to the mean interlayer distance). The ordering degree along *c*-axis is usually characterized by the *P*_3_ parameter [24]. The degree of “disorder” γ = 1 − *P*_3_ is also employed. The structural defects may be evaluated using *c* and *a* lattice parameters. The micro-crystallites may form isotropic or non-isotropic materials depending on their mutual orientation. In general, the conditions of pyrolysis (temperature, growth rate, etc.) allow flexible control of the resulting material.

### 2.2. Turbostratic BN

At highest temperature of pBN production, one can obtain monomodal (only one type of mutual layer orientation) turbostratic (random layer orientation) polycrystalline BN bulks. Random layer lattice structure is an idealization that considers the layers arranged parallel and equidistant, but random in translation parallel to the layer, and rotation about the normal (Inset of Figure 2a). Typical XRD pattern exhibits strongly asymmetric two-dimensional *hk* reflexions. The profile of such line is determined by the crystallite size along the *a*- and *b*-crystallographic directions.

Figure 2b shows the interlayer distance (for turbostratic structure *c* = *d*_001_) of BN with various degree of three-dimensional order (calculated in Reference [25] using the intensities of residual *hkl* reflections according to [24]). For comparison, the data for graphitic carbons are given [26]. The model describing *c*(*γ*) dependence has been previously discussed for graphitic carbon [26] and contains only *γ*^2^ term. In the paper devoted to partially disordered (mesographitic) layered BN [25], both *γ* and *γ*^2^ terms were used for *c*(*γ*) fitting. Although the interlayer interactions in the case of BN are stronger than those between carbon layers, the fit to the equation proposed by Franklin [26] have allowed us to satisfactory describe all the samples except one with *γ* ≈ 0.3. However, the error bar ±δ*γ* may be significant for this point due to the method of calculation (fit of intensity of *102* reflection, respecting the notation of two-layer per unit cell model, that weaken quite fast with the *γ* increase). Our recommended dependence is presented in Figure 2b as a red solid line.

### 2.3. BN Nanoparticles

The BN nanoparticles with layered structure can be produced by, for example, the spray-pyrolysis of borazine, and find many applications (high-temperature lubricants, high-frequency induction furnace materials, etc.). Recently, colloidal synthesis in molten salts has been proposed as a new method of nanoparticles production (nominal composition close to BN, oxygen and carbon are also present in the structure/on the surface) [14]. These particles can be dispersed in water and are potential phosphors with adjustable emission wavelength. No attempts have been made so far to study such precursors at high pressure–high temperature (HPHT) conditions.

cBN (nano)powders can be produced on industrial scale. Subsequently, they can be used for production of BN-based ceramics and composites (with ceramic or metal binders). By direct sintering of nanopowders, the nanostructured ingots can be obtained using some *p-T-time* protocols, however, such ceramics usually show low fracture toughness and wear resistance. These materials, as well as boron nitride colloidal solutions and ultra light aerogels [27], or BN nanosheets [28] will not be discussed here.

## 3. New Advanced B–N Materials

In this section, we will describe recent achievement in materials synthesis by chemical reactions and phase transformations. In all cases the precursors are of great importance.

### 3.1. Boron Subnitrides

First boron subnitride with rhombohedral structure has been mentioned in studies devoted to interaction of boron and nitrogen at high temperatures [29] and to BN chemical vapor deposition on Si substrate [30]. Later, solid-state synthesis of boron subnitride, B_6_N, as a result of chemical interaction between amorphous boron and hBN at 7.5 GPa and ~2000 K has been reported by Hubert et al. [31]. However, a critical analysis of these results and high-pressure studies of B–N interaction with amorphous boron have shown that the evidences were inconclusive for the claims of boron subnitride with truly B_6_O-like or α-B-related structure. Since that time, a number of possible structural candidates have been proposed [32,33], but the structure and composition of reported phases are still to be defined.

**Figure 3:**
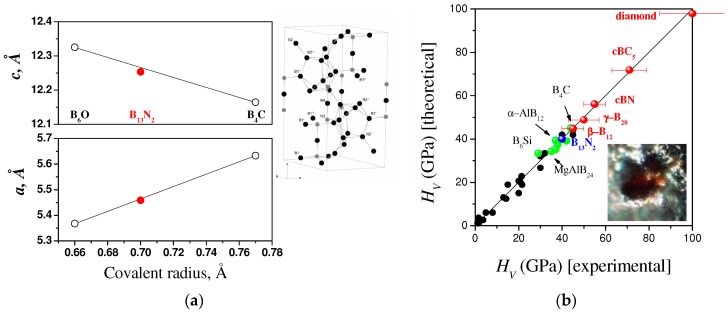
(**a**) Lattice parameters of boron subnitride B_13_N_2_ as compared to other compounds with α-B crystal structure; (**b**) Expected hardness of B_13_N_2_ as compared to other superhard phases.

Chemical interaction and phase relations in the B–BN system at pressures up to 5.3 GPa and temperatures up to 2800 K have been in situ studied by Solozhenko and Kurakevych [18] using X-ray diffraction. New rhombohedral boron subnitride B_13_N_2_ has been synthesized by crystallization from the B–BN melt at 5 GPa. The new phase has been studied by powder X-ray diffraction (conventional and with synchrotron radiation), Raman spectroscopy, high-resolution transmission electron microscopy and electron energy loss spectroscopy. The structure of B_13_N_2_ (Figure 3a) belongs to the *R*-3*m* space group (*a* = 5.4585(8) Å, *c* = 12.253(2) Å) and represents a new structural type produced by the distorted B_12_ icosahedra linked together by N–B–N chains and inter-icosahedral B–B bonds (inset of Figure 3a), how it was shown by XRD and Raman data [18,34,35].

Besides B_13_N_2_, the formation of another boron-rich B–N phase, denoted as “B_50_N_2_” has been observed [18,35]. Its structure has not been resolved so far, but with high probability it belongs to a family of tetragonal boron [36,37]. Similar compound, B_50_N_2_ is known from CVD [38] and electrochemical [39] syntheses. The crystallization of “B_50_N_2_” has been observed only in the B–BN system, while the crystal structure, according to powder XRD should be different. Thus, high pressure is a structure-determining factor, whose role is not understood so far.

According to the semiempirical predictions in the framework of the thermodynamic model of hardness [40], the B_13_N_2_ subnitride is expected to exhibit microhardness of 40 GPa (Figure 3b) comparable to that of commercial polycrystalline cubic boron nitride. Ab initio simulations of hardness show similar results [41]. Tetragonal subnitride B_50_N_2_ has also the hardness approaching the “superhard boundary” of *H*_V_ ≈ 40 GPa [40].

### 3.2. cBN-wBN Nanocomposites

The formation of wBN at moderate synthesis temperatures, required for nanostructuring, unavoidable when using the commercial pBN samples that are characterized by the non-zero degree of three-dimensional ordering *P*_3_. Experiments at 18 GPa and 1900 K starting from powdered commercial turbostratic boron nitride resulted in formation of the superhard aggregated boron nitride nanocomposite cBN/wBN [42]. This nanostructured material shows very high hardness, two-time higher (*H*_V_ ≈ 80 GPa) than that of conventional polycrystalline cBN (*H*_V_ ≈ 40 GPa). This has been attributed to: (1) nanosize effect, which restricts dislocation propagation through the material; and (2) two-phase composition on nano- and subnanometer scale, i.e., to the quantum-confinement hardening of individual crystallites. At the same time, the whole material should have the low thermal stability (wBN-to-rBN transition become possible above 490 K [3]), which should results in a hardness decrease at elevated temperatures. The nanostructured wBN forms in the ordered domains of initial pBN due to the martensitic phase transition; while nano-cBN, in the completely disordered (turbostratic) domains according to more complicated thermally-activated displacive mechanism described in chapter 5.2. Analysis of XRD patterns of wBN [42] reveals non-uniform widening of *hkl* reflections and pronounced asymmetry of some lines. This is indicative of stacking faults and is typical for diamond-like materials [43,44]. Stacking faults additionally confirm the martensitic/displacive nature of wBN formation from ordered regions (the mechanism will be discussed later). The high hardness of the material is explained by the microstructure/hardness coupling known as the Hall-Petch effect [45] (e.g., Vickers hardness *H*_V_ ≈ *d*^−0.5^, where *d* is a mean grain size). Another model implying quantum confinement was also used for BN nanocomposites. In the domain of extremes of mechanical properties of diamond and BN, these phenomena are of great importance [42].

Later, nanotwinned cBN with extreme hardness (*H*_V_ > 100 GPa) has been reported [46], but this result was questioned by other authors [47]. Nano-twinning was also claimed to be responsible for the high stiffness [48] and the absence of inverse Hall-Petch effect that is generally presumed for nanocrystalline sintered ingots.

### 3.3. Nano-cBN

In order to avoid the wBN formation, as in experiments described in the previous section, the monomodal pBN with ideal turbostratic structure (*P*_3_ = 0) was used as a starting material. At 20 GPa the single-phase cBN samples with various grain-size can be synthesized in the 1770–2570 K temperature range [49]. At lower pressures, e.g., ~12 GPa, wBN formation is still possible due to the partial temperature-induced ordering of turbostratic pBN at initial stages of transformation. 

**Figure 4:**
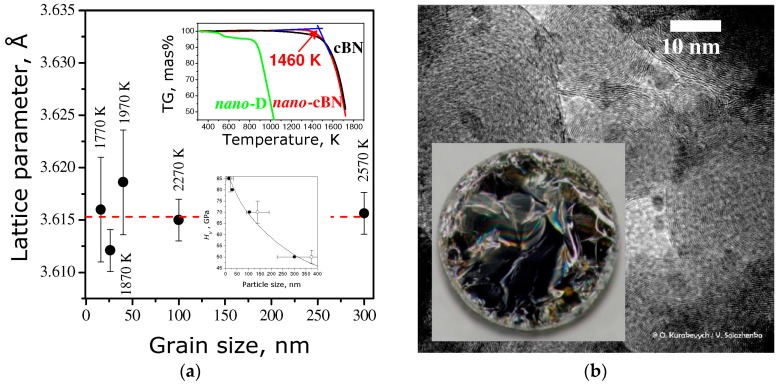
(**a**) Lattice parameters of nano-cBN as a function of grain size; oxidation stability (top inset) and hardness vs. grain size (bottom inset); (**b**) TEM image of nano-cBN grains and the view of the bulk sample recovered from HPHT (inset).

The nano-cBN synthesized at optimal conditions (20 GPa and 1770 K) was the first single-phase non-carbon material with hardness *H*_V_ = 82 (5) GPa (bottom Inset of Figure 4a) exceeding that of polycrystalline diamond. The synthesis of such nanocrystalline material have become possible by applying very high pressure and moderate temperature to pyrolytic boron nitride; while at higher temperatures, due to the bulk/surface diffusion, the polycrystalline cBN forms (*H*_V_ ≈ 40 GPa). The record value of hardness has been achieved by combination of Hall-Petch effect and high grain/intergrain purity inherited from initial compact ingot without free surface (that is usually contaminated). The lattice parameters of nano-cBN samples are very close to the values of high-purity single crystal [50]. The thermal stability of the material remains as high as that of microcrystalline cBN (top Inset of Figure 4a).

## 4. Thermodynamics and Phase Diagrams of the B–N System

The thermodynamics of the B–N and B–BN systems allows understanding the general features of stability, synthesis and mechanisms of transformations. The *p-T* dependence of Gibbs energy *G* is quite important for that. The “thermal part” of *G* can be established by the temperature dependence of heat capacity, while for the “pressure part” at given temperature, one needs reliable *p-V-T* equations of state.

### 4.1. p-V-T Equations of State of BN

The knowledge of the *p-V-T* equations of state (EOS) is quite important for thermodynamic analysis of the BN and B–BN systems under pressure. To fit experimental data we have used the integrated form of the Anderson–Grüneisen equation [51], described in our previous work [52] and successfully applied to fit the data for compounds with various chemical bonding, both solids and liquids [53,54,55,56]:

(1) $$V(p,T)={\left[V{\left(0,T\right)}^{-\delta_{T}}+V{\left(p,300\right)}^{-\delta_{T}}-V{\left(0,300\right)}^{-\delta_{T}}\right]}^{-1/\delta_{T}}$$

where thermal expansion (i.e., *V*(*0*,*T*) at 0.1 MPa) and isothermal compression (i.e., *V*(*p*,300) at 300 K) can be presented in any analytical form, e.g., polynomial

*V*(0,*T*) = *V*(0,300) [1 + *a* (*T* − 300) + *b* (*T* − 300)^2^] (2)

and Murnaghan [57] (or any other) equation of state

(3) $$V(p,300)=V(0,300){\left(1+{B^{\prime }}_{0}p/B_{0}\right)}^{-1/B_{0}}$$

Finally, a set of parameters needed to describe an EOS using Equations (1)–(3) is *V*_0_ = *V*(0,300), *B*_0_, *B*_0_’, *a*, *b* and *δ*_T_. All these values for B–N phases are tabulated in Table 1.

The calculation of the Gibbs energy under such conditions is possible by using the heat capacity data at 0.1 MPa and EOS data using equation

(4) $$G(p,T)=G(0,T)+\int_{0}^{p}V(\pi ,T)d\pi =G(0,T)+\int_{0}^{p}{\left[V{\left(0,T\right)}^{-\delta_{T}}+V{\left(\pi ,300\right)}^{-\delta_{T}}-V{\left(0,300\right)}^{-\delta_{T}}\right]}^{-1/\delta_{T}}d\pi$$

**Figure 5:**
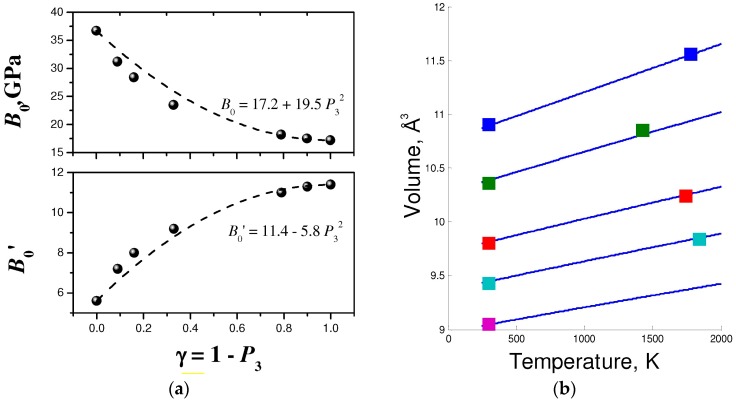
Equations of state of layered BN forms: (**a**) Bulk moduli and their pressure derivatives as a function of structural disorder along *c*-axis [58]; dashed lines show the model with only *P*_3_^2^ term by analogy to lattice parameter dependence (Figure 2b); and (**b**) isobars (from top to bottom: 0.1 MPa, 2 GPa, 5.2 GPa, 8 GPa and 12 GPa) for hBN, experimental data from [59], adjusted to the *p-V-T* EOS.

**Figure 6:**
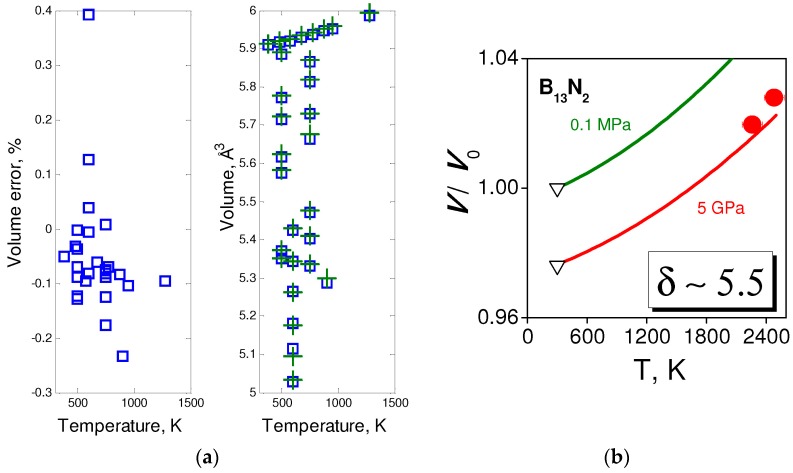
*p-V-T* equations of state: (**a**) cBN, experimental data from [60], adjusted to *p-V-T* EOS; and (**b**) B_13_N_2_, experimental data from [62], adjusted theoretical values for EOS parameters.

Figure 5 and Figure 6 show experimental vs. fitted data for the *p-V-T* equations of state of various forms of BN and for B_13_N_2_. The parameters of equations of state are given in Table 1. Figure 5a indicate the dependence of bulk moduli *B*_0_ and their pressure derivatives *B*_0_’ on the structural disordering *γ* along the *c*-direction. This dependence has been first observed by Solozhenko and Solozhenko in 1996 [58] and explained large discrepancies in the previous experimental data, where structural ordering has not been taken into account. The *p-V-T* equation of state of hBN (*γ* ≈ 0) has been reported in Reference [59]. The data and its fit to the EOS (1–3) are presented in Figure 5b.

Figure 6a represents the *p-V-T* measurements for cBN reported by Datchi et al. in 2007 [60], as well as fit to Equations (1)–(3) and uncertainties. In the case of B_13_N_2_, only limited number of experimental points is available. Figure 6b shows the experimental data extracted from HP [61] and HPHT [62] measurements, and fit combined with semiempirical modeling [53]. 

### 4.2. Equilibrium Phase Diagram of BN

A first version of phase *P*,*T*-diagram for boron nitride was proposed by Bundy and Wentorf in 1963 [65] based on Wentorf’s experimental data on hBN-to-cBN conversion [2,65] and data on hBN melting under high pressures [66]. Later, Corrigan and Bundy [67] improved the diagram of 1963 by analogy with the carbon diagram, and this version of BN phase diagram was generally accepted up to the late 1980s.

**Figure 7:**
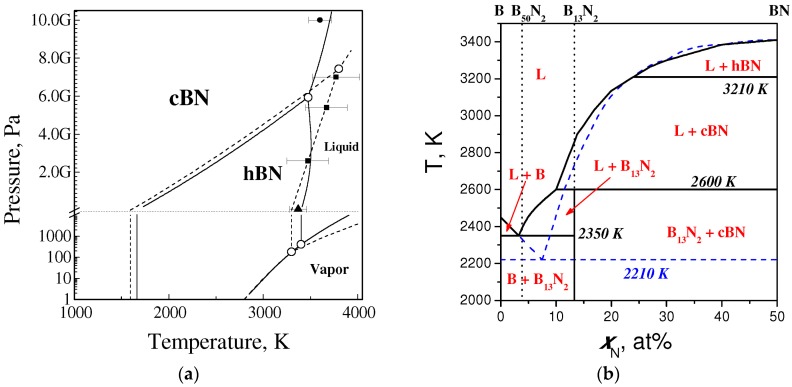
(**a**) Equilibrium phase *P*,*T*-diagram of boron nitride (solid lines—the refined diagram [69], dashed lines—diagram of 1988 [68]). Solid squares show Wentorf's data on hBN melting [66], solid circle is the melting point of cBN at 10 GPa [76], and solid triangle is hBN melting point at 50 MPa [74]; (**b**) Phase diagram of the B–BN system at 5 GPa [62]. Solid and dashed lines show the equilibrium and metastable phase diagrams respectively.

In 1988, a new equilibrium phase diagram of boron nitride (Figure 7a) was suggested in the framework of thermodynamic approach [68]. This diagram differs drastically from the previous ones [66,68], thus rejecting the assumed analogy of phase diagrams for carbon and boron nitride.

Later phase equilibrium lines for all BN polymorphs as well as lines of equilibria of crystalline phases with vapor and liquid have been calculated up to 10 GPa and 4000 K [69] based on experimental data on thermodynamic properties [70] and compressibility and thermal expansion [63,71,72]. From hBN melting entropy of 25 J/(mole·K) [73] and melting temperature of 3400 K at 50 MPa [74], the melting enthalpy of hBN was estimated as 85 kJ/mole. From the value of the initial slope (70 K/GPa) of Wentorf’s melting curve for hBN [66], the molar volume of the liquid BN under standard conditions was found to be 12.42 × 10^−6^ m^3^/mole. To describe the compressibility of liquid boron nitride, Murnaghan's equation of state was used with the bulk modulus *B*_0_ = 13.3 GPa and its pressure derivative *B*_0_′ = 2, by analogy with Gustafson’s approach to describing the molar volume of liquid carbon [75].

The cBN⇆hBN equilibrium line intersects the temperature axis. Both nucleation of cBN and its crystal growth were observed without high-pressure techniques [10], which illustrate the thermodynamic stability of cBN at ambient pressure and confirm the thermodynamic calculations. No regions of wBN and rBN thermodynamic stability have been found.

The refined equilibrium phase diagram of boron nitride [69] is shown in Figure 7a. As compared with the 1988-version [68], the hBN ⇆ cBN equilibrium line is displaced by 60 K towards higher temperatures. The higher compressibility of the liquid phase with respect to graphite-like hexagonal boron nitride first causes the slope of the hBN melting curve to increase with pressure up to 3.4 GPa and then the slope becomes negative. The intersection of the calculated hBN melting curve with hBN ⇆ cBN equilibrium line defines the hBN–cBN–liquid triple point at 3480 K and 5.9 GPa.

Wentorf’s experimental data on hBN melting [66] are in reasonable agreement with the calculated hBN melting. The experimental point at 6.7 GPa obviously corresponds not to hBN but to cBN melting. The experimental cBN melting point at 10 GPa [76] is in a reasonable agreement with the calculated melting curve of cBN.

### 4.3. Phase Diagrams of the B–BN System

Chemical interaction and phase transformations in the B–BN system at pressures up to 5.3 GPa and temperatures up to 2800 K have been in situ studied using synchrotron X-ray diffraction and quenching [62]. It has been found that only one thermodynamically stable boron subnitride, namely, B_13_N_2_, exists in the system. At 5 GPa, B_13_N_2_ melts incongruently at 2600 K and forms eutectic equilibrium with boron. The equilibrium phase diagram of the B–BN system at 5 GPa (Figure 7b) is characterized by the following nonvariant equilibria: L + BN ⇆ B_13_N_2_ of peritectic type at 2600 K; L ⇆ β-B + B_13_N_2_ of eutectic type at 2300 K; and L ⇆ β-B + BN metastable eutectic at 2120 K that assures the appearance of the liquid phase, from which B_13_N_2_ crystallizes [62].

By combination of above-mentioned thermodynamic studies of B–BN phase diagram with previous results for the B–B_2_O_3_ and BN–B_2_O_3_ binary systems [77], the B–BN–B_2_O_3_ phase diagram at 5 GPa has been constructed [78].

## 5. Kinetics of BN Phase Transformations

Understanding of the kinetics of phase transformations between BN polymorphs is a key point in understanding of the formation of a wide range of materials, from nanostructures to large-grain polycrystalline bulks. Kinetic aspect of BN crystallization from melts/fluids have been previously studied in situ [63,79,80,81,82], which shed light on the existence of the threshold pressure for cBN crystallization from melts of systems traditionally used for its commercial synthesis (hBN–Mg_3_N_2_, hBN–Li_3_N, etc.); while for direct transformations, there remain many controversies that will be addressed. In this section, we will analyze the available data from previous studies [65,83,84], as well as our in situ data obtained under experimental conditions previously explored in other kinetic studies (e.g., ZnO [85]).

### 5.1. Kinetics of Direct hBN-to-cBN Phase Transformation

Direct phase transition hBN → cBN was first observed by Bundy and Wentorf in 1963 [66]. Next, different authors made the attempts to study the kinetics of the process in quenching experiments using hBN, tBN and pBN as starting materials, but no agreement in kinetics parameters has been achieved [67,83,84]. The estimated values of the activation energy differ from each other by 400 kJ/mole, and no correlation between the characteristics of initial BN material and kinetics was found. To analyze the reported and our data we have used Avrami’s equation [86] with approximation of full transformation for extended volume model [87]:

(5a,b) $$-\ln\left(1-\alpha \right)={\left(k\cdot t\right)}^{r}\;\mathrm{or}\;-\ln\left(1-\alpha \right)={\left(\int_{T_{0}}^{T}\frac{k\left(\tau \right)}{\beta }\cdot d\tau \right)}^{r}$$

with simple Arhenius-type dependence of kinetic constant [88]

(6) $$k\left(T\right)=e^{z-\frac{E_{A}}{R\cdot T}}$$

In our experiments, we have studied in situ the direct phase transitions tBN → hBN → cBN by synchrotron X-ray diffraction at 6.5 GPa and temperatures up to 2000 K. Such experiment is rather precise, and allowed us to establish the temperature and pressure during the process, and to obtain the kinetic curve using the same sample. Non-isothermal conditions make possible to calculate the activation energy from the results of just one experiment. In the Avrami Equation (5) we fixed the value *r* = 1 (i.e., in the suggestion that two-dimensional diffusion on surface grains is the process that limit grain growth, similar to most other direct phase transitions [83,84,85,87]).

At 6.5 GPa, in the course of tBN heating in high-pressure cell with in situ probing with synchrotron radiation we have observed the rise of hBN lines and disappearance of the broad lines of the starting turbostratic phase. At 1600 K the transformation of tBN into hBN seems to be completed. The well-known Tamman rule states that the solid diffusion intensifies at a temperature corresponding to a half of absolute melting temperature [89]. The Tamman temperature of hBN is *T*_T_ ≈ 1650 K (a half of *T*_m_ = 3300 K at 6.5 GPa, according to the phase diagram of BN [69]), which is almost the same that the onset temperature of the observed transformation, *T*_t_ ≈ 1620 K. This fact reveals the critical role of solid diffusion in direct BN phase transformation (e.g., as in the case of ZnO previously observed in situ [85]) in accordance with HPHT diffusion-reconstructive mechanism [44,90].

**Figure 8:**
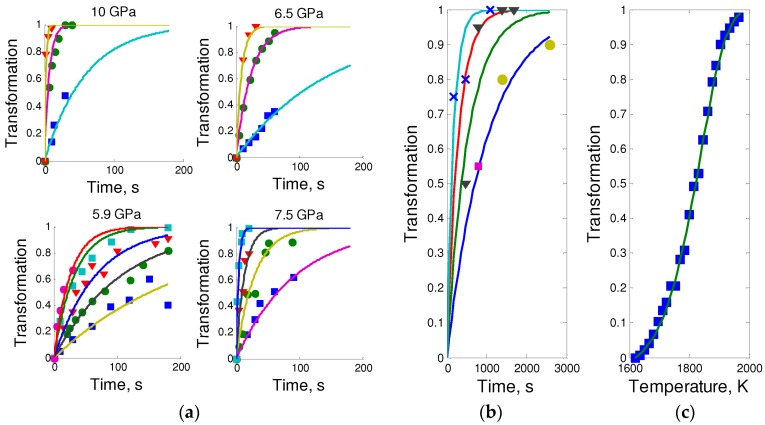
Kinetic curves for hBN-to-cBN transformation (all theoretical curves calculated using Equations (5) and (6)): (**a**) Top: isothermal kinetic data from [83] for pure pyrolytic hBN; bottom: isothermal kinetic data from [84] for hBN of technical grade; (**b**) isothermal kinetic data from [67] for pure hBN; and (**c**) non-isothermal kinetic data (linear heating, heating rate β = 10 K/s) from our in situ observations for pure hBN obtained by thermally induced ordering of tBN.

Figure 8 shows the results of the fit of our kinetic data in comparison with results of previous works. Similar fitted parameters were obtained in the case of pure crystalline hBN, while for the technical grade hBN the activation energies were 30%–60% lower, (most probably due to the presence of boron oxide that is known to promote this phase transformation). The early studies of transformations of pure hBN led to very high values of activation energy, but the method to extract it was quite ambiguous, and our fit (Figure 8a) shows that the data are very consistent with our estimations of activation energy, i.e., ~320 kJ/mol rather than ~600 kJ/mol [67]. The activation energy of the surface self-diffusion is *E*_D_ = 3.36 keV/atom, which is a typical value for vacancy migration in strong covalent solids like carbon allotropes.

Here we should note that tBN shows similar behavior as it has been found for pyrolytic BN samples [91]. No visible transformation at temperatures below diffusion onset, and fast transformation at higher temperatures. Up to 10 GPa, the minimal grain sizes of fully transformed samples are always higher then 100 nm. 

**Figure 9:**
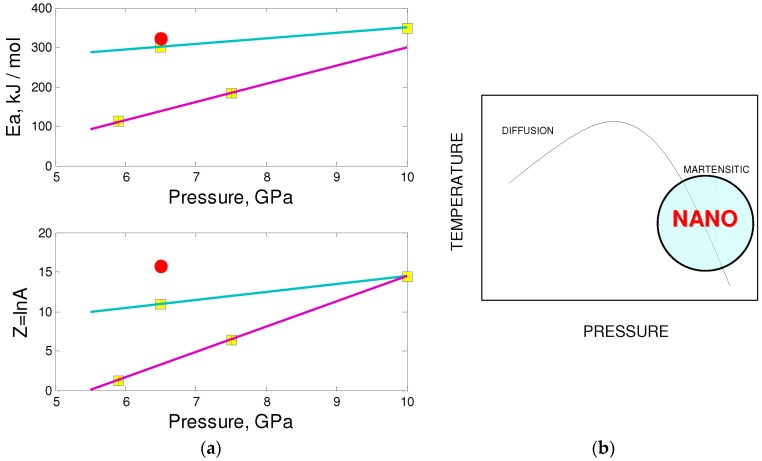
Kinetic parameters for hBN-to-cBN transformation: (**a**) Activation energy (top) and logarithm of pre-exponential factor (bottom) as function of pressure (open squares for pyrolytic hBN, crossed squares for hBN of technical grade, open circles for pure hBN obtained by thermally induced ordering of tBN); (**b**) Pressure–temperature diagram indicating the relative position of diffusive and displacive (martensitic) mechanisms. The latter favor the formation of nanostructures.

Although at pressures below 10 GPa the direct transformation of layered structures into dense allotropes occur only in the framework of diffusional mechanism at quite high temperatures, the pressure increase may lead to transformation into dense polymorphs (sometimes irreversible, depending on the initial form of boron nitride). Such transformations are called reconstructive (martensitic) and typically give rise to stacking faults and particular crystallographic relationships between initial and final phases [90] (Figure 9b). Moderate heating allows stabilizing of high-pressure phases at noticeably lower temperatures, when required for diffusion. In the case of ordered hBN and rBN, the systematic displacements and buckling of layers were observed by different methods [92,93]. This mechanism is more promising for creation of nanostructures, although previously it has been reported only for the transformation of polycrystalline graphite into nano-diamond at HPHT conditions [21].

### 5.2. Mechanism of Direct tBN-to-cBN Phase Transformation: Reconstructive Features and Thermal Activation

As mentioned above, below 10 GPa the HPHT behavior of turbostratic structures is pretty close to the ordered counterparts and is determined by diffusion. However, at higher pressures the situation is more complex. The mechanism of the HPHT transformation of turbostratic graphite-like phases into diamond-like structures has been first suggested [7] in the framework of the reversible diffusionless transformation of the initial turbostratic structure into a high-pressure phase formed by close-packed buckled layers having a diamond-like structure [94,95,96]. The proposed crystallographic mechanism allows explaining the fact that the synthesis of the cubic phases (either stable or metastable) is possible only under very high pressures (≥20 GPa) and at relatively low temperatures, when the role of the diffusional processes is not very important [7,49,96]. The in situ X-ray diffraction and Raman scattering experiments in diamond anvil cells have shown that in the course of compression at room temperature, all turbostratic graphite-like B–C–N phases show similar behavior that is indicative of the phase transformation associated with a discontinuous change of interlayer distances [96]. The mechanism including the buckling (sp^2^-to-sp^3^ transition) and change of the mutual orientation of layers has been established by the crystallographic simulation [96]. At pressures about 25 GPa, the initial turbostratic phase passes into the disordered high-pressure phase consisting of buckled diamond-like layers. At room temperature, the transformation is fully reversible and the formation of covalent bonds between the layers does not occur. After heating, the layers start rearranging without significant grain growth. Thus, at moderate temperature, one can obtain nanostructured diamond-like phases, as was already observed in the case of diamond-like BC_5_ [7] and described above for nano-cBN [49]. The proposed crystallographic mechanism allows explaining the fact that the synthesis of the metastable cubic phases is possible only under very high pressures (≥20 GPa) and at relatively low temperatures, when the diffusional processes are hindered [7,49,96].

## 6. Conclusions

Extreme pressure–temperature conditions are powerful and promising tool for: (i) synthesis of novel phases via chemical interaction; and (ii) grain-size control during direct solid-state phase transformations. The simultaneous variation of pressure and temperature makes it possible to combine different nucleation, growth and aggregation regimes with high flexibility, and, therefore, to go deep into design of new advanced materials.

## Figures and Tables

**Table 1:** Parameters of the *p-V-T* equations of state of BN polymorphs and polytypes.

B–N phase	Fitted Parameters ^1^	Fixed Parameters ^1^
cBN [60]	*B*_0_ = 390; *B*_0_’ = 3.35; *δ* = 3; *a* = 1 × 10^−6^; *b* = 5 × 10^−9^	
wBN [63]		*B*_0_ = 375; *B*_0_’ = 4.9; *δ* = 3; *a* = 1 × 10^−6^; *b* = 5 × 10^−9^
nano-cBN [64]		*B*_0_ = 375; *B*_0_’ = 2.3; *δ* = 3; *a* = 1 × 10^−6^; *b* = 5 × 10^−9^
hBN [59]	*δ* = 2.5; *a* = 4.1 × 10^−5^	*B*_0_ = 36.7; *B*_0_’ = 5.6; *b* = 0
tBN [58]		*B*_0_ = 17.2; *B*_0_’ = 11.4
B_13_N_2_ [53]		*B*_0_ = 200; *B*_0_’ = 4; *δ* = 5.5; *a* = 1.4 × 10^−5^; *b* = 5 × 10^−9^

^1^ The units: *B*_0_ in GPa, *B*_0_′ and δ without units, *a* in K^−1^, *b* in K^−2^.
